# Review: Precision Medicine Approaches for Genetic Cardiomyopathy: Targeting Phospholamban R14del

**DOI:** 10.1007/s11897-022-00558-x

**Published:** 2022-06-14

**Authors:** Frederik E. Deiman, Nils Bomer, Peter van der Meer, Niels Grote Beverborg

**Affiliations:** Department of Cardiology, University Medical Center Groningen, University of Groningen, UMCG Post-zone AB43, PO Box 30.001, 9700 RB Groningen, The Netherlands

**Keywords:** Heart failure, Genetic heart disease, Congenital heart disease, Phospholamban, Phospholamban R14del-associated cardiomyopathy, Precision medicine

## Abstract

**Purpose of Review:**

Heart failure is a syndrome with poor prognosis and no curative options for the majority of patients. The standard one-size-fits-all-treatment approach, targeting neurohormonal dysregulations, helps to modulate symptoms of heart failure, but fails to address the cause of the problem. Precision medicine aims to go beyond symptom modulation and targets pathophysiological mechanisms that underlie disease. In this review, an overview of how precision medicine can be approached as a treatment strategy for genetic heart disease will be discussed. PLN R14del, a genetic mutation known to cause cardiomyopathy, will be used as an example to describe the potential and pitfalls of precision medicine.

**Recent Findings:**

PLN R14del is characterized by several disease hallmarks including calcium dysregulation, metabolic dysfunction, and protein aggregation. The identification of disease-related biological pathways and the effective targeting using several modalities, including gene silencing and signal transduction modulation, may eventually provide novel treatments for genetic heart disease.

**Summary:**

We propose a workflow on how to approach precision medicine in heart disease. This workflow focuses on deep phenotyping of patient derived material, including in vitro disease modeling. This will allow identification of therapeutic targets and disease modifiers, to be used for the identification of novel biomarkers and the development of precision medicine approaches for genetic cardiomyopathies.

## Introduction

Heart failure (HF) is a disease with a high mortality rate and increased prevalence with age [1, 2]. HF is characterized by an insufficiency of cardiac output. The biological events leading to HF are generally accepted to first manifest at the level of the cardiomyocyte, wherein dysregulated calcium (Ca^2+^) plays an essential role [3]. The current treatment options for HF target the activated neurohormonal system, using renin-angiotensin-aldosterone inhibitors, beta blockers, and sodium-glucose-transporter 2 inhibitors, but not intracellular calcium regulation [4]. Although these drugs improved the survival of HF in the last decades, the prognosis is still very poor with a 5-year survival rate of less than 50% [2, 5]. This poor prognosis demonstrates the necessity of novel treatment strategies to fight HF. Precision medicine is a rapidly developing treatment option as it allows us to alleviate heart disease, not in a one-size-fits-all manner, but by a specific therapy that targets pathophysiological mechanisms that underlie disease. This can for instance target a specific etiology, phenotype, comorbidity, or genetic mutation [6]. In this review, we will summarize the development regarding precision medicine for genetic cardiomyopathies over the last decade, with a special focus on Phospholamban (PLN)-R14del-associated cardiomyopathy.

## Genetic Cardiomyopathy

Genetic cardiomyopathies are disorders of the myocardium that arise due to the genetic background of an individual [7]. The known genetic cardiomyopathies are usually monogenetic and inherited in an autosomal dominant pattern [8], making them potential therapeutic targets for precision medicine. The mutations can lead to different types of heart disease including congenital heart disease, hypertrophic cardiomyopathy (HCM), dilated cardiomyopathy (DCM), and arrhythmogenic cardiomyopathy (ACM) [9]. Over 1000 pathogenic mutations have been identified over the previous few decades and mutations in certain structures are known to lead to certain types of heart disease [9]. As effective Ca^2+^ cycling is essential in cardiomyocytes, mutations affecting Ca^2+^-related genes are known to cause both DCM and ACM [9]. Therefore, Ca^2+^ cycling could be a highly potent treatment target for patients with these mutations, but also for patients with other HF etiologies where dysregulated Ca^2+^ metabolism is commonly found. Changes in Ca^2+^ cycling are a key feature of HF pathophysiology. The changes are known to impact contraction of the heart and are associated with arrhythmias, adverse remodeling, and impaired cardiac performance [10, 11, 12].

## PLN Cardiomyopathy

**Fig. 1:**
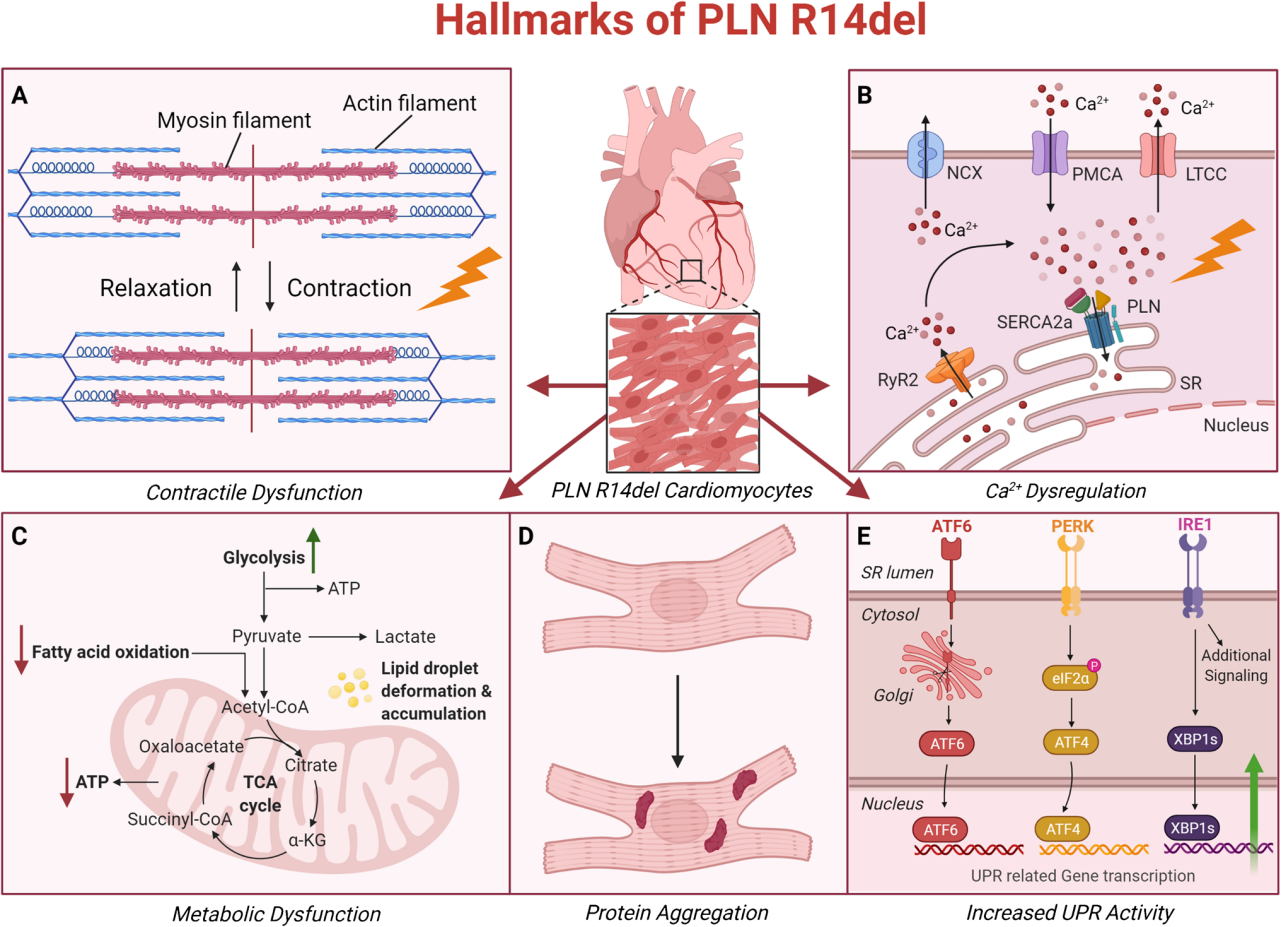
Hallmarks of PLN R14del. Five hallmarks of PLN R14del that have been identified on a cardiomyocyte level. **A** Contractile dysfunction, **B** Ca^2+^ dysregulation, **C** metabolic dysfunction, **D** protein aggregation, and **E** increased UPR activity. Created with BioRender.com

Ca^2+^ is a ubiquitous intracellular second messenger that has a key role in cardiomyocyte fitness [13]. It has an essential role in energy metabolism, proliferation, apoptosis, and cardiomyocyte contraction [13, 14]. Ca^2+^ regulates cardiomyocyte contraction by binding to myofilament proteins in the cytosol. This allows shortening of the sarcomeres that are required for force generation [13]. Important players herein are sarco(endo)plasmic reticulum Ca^2+^-ATPase 2a (SERCA2a) and PLN as they regulate re-uptake of Ca^2+^ into the sarcoplasmic reticulum, the intracellular Ca^2+^ storage [13, 15]. PLN is a transmembrane phosphoprotein of the SR that regulates SERCA2a activity depending on phosphorylation state (Fig. 1b) [15]. The phosphorylation state dictates whether PLN binds to and regulates the activity of SERCA2a. When dephosphorylated, PLN is an inhibitor of SERCA2a, thereby inhibiting the Ca^2+^ reuptake from the cytosol into the SR. When phosphorylated by cAMP-dependent protein kinase (PKA), due to for example B-adrenergic receptor pathway signaling, PLN dissociates from SERCA2a, thereby activating SERCA2a, increasing Ca^2+^ reuptake into the SR, and increasing relaxation rate [15]. Due to this, modulating PLN is expected to have beneficial properties in heart disease. This is because excessive inhibition of SERCA2a by PLN is said to be an important player in the pathogenesis of HF. In addition, studies have demonstrated that cardiac function can be improved by genetically modifying PLN [16, 17].

PLN mutants, such as PLN R14del, R9C, and L39X, are associated with severe cardiomyopathy that can lead to HF [18, 19, 20]. Among these mutants, the R14del mutation in PLN is the most prevalent in cardiomyopathy patients in the Netherlands and is found in 15% of idiopathic DCM and 12% of ACM patients [18, 21]. Until now, there is no specific treatment available for patients with PLN R14del-associated cardiomyopathy. Patients suffering of PLN R14del-associated cardiomyopathy are treated like any other type of patients with HF, though the etiology is very different from standard HF (Fig. 1). PLN R14del-associated cardiomyopathy is a very detrimental disease with a poor prognosis [22], and a large group of the patient population ultimately find themselves in need of a left ventricular assist device or cardiac transplantation. The clinical phenotype of PLN R14del-associated cardiomyopathy is characterized by cardiac fibrosis, PLN protein aggregation, fibrofatty replacement, and decreased ECG potentials [21, 23]. Until recently, the PLN R14del mutation was thought to be a super inhibitor of SERCA2a [24, 25], but experimental evidence is conflicting as some studies find no inhibition of SERCA2a by PLN R14del [26, 27•]. On a cardiomyocyte level, PLN R14del is known to impact cardiomyocyte function greatly. Studies have demonstrated that the R14del mutant leads to PLN protein aggregation [23], increased unfolded protein response activity [28•, 29•], calcium dysregulation [24, 27•, 29•, 30•], and contractile [24, 28•, 30•] and metabolic dysfunction [28•, 29•, 31] (Fig. 1). These hallmarks are also common in other etiologies of HF. PLN R14del-associated cardiomyopathy is an excellent example of a genetic disease in urgent need of deep phenotyping to elucidate the causal biological mechanisms of disease and finding a precision medicine for this cardiomyopathy population. PLN R14del furthermore lends itself greatly to precision medicine due to the seemingly monogenetic nature and the relatively large number of carriers of the disease that have been identified, specifically in the Netherlands, which can help in uncovering disease etiology.

## Modalities of Precision Medicine

### Gene Editing

Using gene editing strategies, the core of the problem in genetic disease can be addressed, the mutation [32]. Several genome editing strategies exist, among which clustered regularly interspaced short palindromic repeats (CRISPR) and CRISPR-associated protein 9 (CRISPR-Cas9) [33] is the golden standard. Alternatives include transcription activator-like effector nuclease (TALEN) [34] and zinc finger nuclease (ZFN) [35], and the latter of which has been used in humans in small scale [36], but both have fallen into disuse with the development of CRISPR-Cas9. Gene editing strategies are capable of changing the genome by either correcting a mutation or introducing a new sequence. CRISPR-Cas9 has the ability to precisely remove or modify the area covering the R14del mutation from the genome of patients with PLN R14del-associated cardiomyopathy. This allows the use of gene editing to have great therapeutic potential for the treatment of genetic disease in the future. However, the use of gene editing in humans is largely controversial, due to the occurrence of non-target mutations, side effects, genetic mosaicism, and ethical concerns [37, 38]. CRISPR-Cas9 is a constantly improving and evolving field. With the introduction of base and prime editing, the side effects of standard CRISPR-Cas9 may be circumvented. In base editing, mutations can directly be inserted within the DNA without inducing double-strand breaks that are a cause of side effects and common in standard CRISPR-Cas9 [39, 40]. Alternatively, in prime editing, new genetic information can be directly inserted into a specified DNA site. This is achieved using a guide RNA that targets a specific site and encodes the desired edit. In this way, prime editing has a similar efficiency to other CRISPR-Cas9 strategies with fewer byproducts and lower off-target editing [41].

CRISPR-Cas9 has shown to be a promising future treatment strategy as genetic forms of sickle cell disease and β-thalassemia have successfully been treated ex vivo [42•]. In this way, a complete controlled trial has been performed that has shown to be efficient, effective, and safe for the treatment of monogenic disease with no off-target effects in humans [42•]. Additional clinical trials involving gene editing are ongoing for the treatment of cancer and inherited hematologic, metabolic, and eye disorders [43]. In vivo studies using gene editing in relation to the heart are scarce, but have been successful in the treatment of amyloidosis [44], atherosclerosis [45, 46], and Duchenne muscular dystrophy [47•]. In one of these studies, amyloidosis was ameliorated through a single dose of NTLA-2001 in a group of patients with hereditary amyloidosis [44]. NTLA-2001 is a gene-editing therapeutic that prevents production of functional TTR protein using a CRISPR-Cas9 system. NTLA-2001 successfully reduced serum TTR levels, the main culprit in this amyloidosis, with only mild adverse events [44]. Atherosclerosis was ameliorated in vivo using gene editing in the liver in two separate studies. In the first, disruption of PCSK9, a protein that functions as an antagonist to the LDL receptor, reduced blood cholesterol levels without any known adverse events [45]. In the second, correcting a mutation in the LDLR gene that is known to be a main cause of familial hypercholesterolemia showed to reduce total cholesterol and aortic plaque formation, without any reported adverse events [46]. Lastly, Duchenne muscular dystrophy was ameliorated in vivo by repairing dystrophin using CRISPR-Cas9, thereby improving muscle dysfunction in both skeletal muscle fibers and cardiomyocytes [47•]. Future research will show whether and how gene editing can be applied to help patients with genetic heart disease. The challenges here would be making a gene editing strategy that targets the heart efficiently, clears the mutation, and has no off-target effects.

### Selective Gene Silencing

There are several ways in which silencing of genes can be achieved. Antisense oligonucleotides (ASOs), synthetic miRNA (smiRNA), short hairpin (shRNA), small interfering (siRNA), and single-stranded siRNA (ss-siRNA) are gene silencers that are capable of degrading mRNA and thereby repress protein translation [48, 49]. Selective silencing of only one copy of a gene may be advantageous over non-selective silencing of both copies. With regard to PLN R14del, silencing both mutated and normal copies of PLN may be therapeutically beneficial [16, 17]. The recessive PLN p.Glu2Ter mutation however, which is known to prevent PLN synthesis, associates to severe DCM and heart failure [50]. Therefore, preservation of normal PLN expression is probably essential in humans as a complete PLN knockout is likely harmful. Gene silencers can be made allele-specific to effectively and selectively silence the mutant PLN transcript by targeting the R14del site specifically. PLN siRNAs have improved contractile function in failing human cardiomyocytes [51]. In addition, in rat cardiomyocytes with a decreased SERCA2a protein level, the PLN siRNA was able to restore cardiomyocyte Ca^2+^ uptake [52]. siRNAs targeting PLN are known to possess beneficial properties in vivo as well as in vitro models of HF [51, 52, 53]. Whether PLN siRNAs will be capable of alleviating, PLN R14del remains to be studied. A gene silencing modality that has recently shown to be very promising for the treatment of R14del is the ASO. In a homozygous PLN R14del-associated cardiomyopathy mouse model, we have shown that the PLN-ASO prevents PLN protein aggregation and cardiac dysfunction, as well as leading to a 3-fold increase in survival rate [54•]. Later, it was demonstrated that the PLN-ASO not only halts disease progression but is also capable of partially reversing the disease phenotype, by eliminating PLN protein aggregates, but it failed to resolve existing tissue damage [55•]. Interestingly, the PLN-ASO also showed beneficial effects on cardiac function in other etiologies of experimental HF, such as DCM and ischemic cardiomyopathy [54•]. In this way, gene silencing of PLN is known to possess beneficial properties in both PLN r14del as well as general HF, allowing it to be a promising treatment strategy for PLN r14del-associated cardiomyopathy and HF.

### Gene Expression Modulators

Factors that are capable of modulating the activation of transcription factors are commonly called modulators. They play an essential role in gene expression responses based on the signals a cell receives [56]. Knowing what biological pathways are capable of inducing or repressing PLN expression may be potentially interesting pathways to modulate using small molecules. For example, modulating gene expression of PLN using zinc-finger protein transcription factors improved function of the failing heart by increasing calcium re-uptake kinetics and improving contractile function both in vitro and in vivo [57]*.* Identifying modulators of PLN and their mode of action, and their downstream targets may give rise to novel therapeutic targets that may be of interest to modulate PLN. Gene expression modulators that are known to modulate PLN (e.g., GSK3 [58, 59], TGF-β [60], hypoxia [61], tafazzin [62]) can be targeted using, for example, small molecules or intrabodies. So far, modulation of these pathways has not been studied in relation to PLN R14del. Studying the activity of these gene expression modulators in the R14del population disease spectrum may be of interest as they may play an important role in disease penetrance. The biological processes underlying the formation of PLN are still poorly understood, but deserve attention as they may play an important role in the development of disease [16, 17].

### Intrabodies

Antibodies are an excellent tool to, with high specificity and affinity, bind and modulate antigens. Intrabodies serve as an alternative to antibodies, whereas antibodies can be designed to specifically bind to antigens outside of the cell [63, 64]. Intrabodies are antibodies that are designed to be expressed within the cell and be used to specifically target an antigen at various compartments of the cell including the nucleus, cytosol, endoplasmic reticulum, mitochondria, and vesicles [63, 65]. Intrabodies may be of benefit in R14del in several manners. They could be developed to impair the binding of PLN to SERCA2a, or they could be used to impair signal transduction routes that play a pivotal role in R14del disease development. The specificity of intrabodies can include post-translational modifications, allowing for a more precise therapeutic to be used to alter cellular physiology [64]. The potential of intrabody use as a therapeutic has been shown in neurodegenerative disorders [66, 67], infectious disease [68, 69], and cancer [70, 71]. Intrabodies are understudied in heart disease. The challenges that intrabodies face are cellular specificity. In order to ameliorate R14del, intrabodies need to be expressed within cardiomyocytes using, for example, target cell directed gene delivery. Being capable of tailoring intrabodies to be expressed in specific cells with the right duration, to interact with proteins and biological pathways of interest, allow intrabodies to be a promising drug of the future.

### Small Molecules

Organic compounds that are capable of modulating molecular pathways are called small molecules. They include lipids, monosaccharides, second messengers, and other natural products and metabolites [72]. To identify small molecules that are able to alleviate PLN R14del, a high-throughput screening of compounds can be performed. High-throughput screening is one of the leading manners in which promising molecules for drug development can be discovered [73]. When therapeutic targets have been identified, a high-throughput screening can be performed to discover novel compounds that are capable of modulating a therapeutic target [73]. The novel compounds can then be further studied for validation and characterization. Promising molecules that have shown to possess beneficial properties in PLN R14del are istaroxime and binding immunoglobulin protein inducer X (BiX) [28•, 30•]. In an in vivo model of R14del in zebrafish, that is characterized by changes in contractility and in the action potential. Istaroxime has shown to improve Ca^2+^ dysregulation and normalize the action potential. The relevance and translatability of this research in this disease model to the human setting remains to be studied [30•]. However, it was shown that PST3093, an istaroxime metabolite, does not correct Ca^2+^ dysfunction of PLN R14del hiPSC-CM [27•]. In a human in vitro model of PLN R14del, BiX was identified as a promising drug to treat R14del [28•]. In this study, PLN R14del was modeled using hiPSC-CMs including an isogenic control. Here, they found that PLN R14del hiPSC-CMs possessed contractile dysfunction and that the unfolded protein response (UPR) pathway was activated [28•]. When the UPR was further inhibited using selective gene silencing strategies, the contractile dysfunction was increased. Further activating the UPR using BiX improved contractility in both 2-dimensional as 3-dimensional cultures of R14del hiPSC-CMs, shedding light on the role of the UPR in R14del disease pathology and revealing and interesting therapeutic target that can be modulated using small molecules [28•].

### Signal Transduction Modulation

Cellular signaling is a property of all cells and controls cellular homeostasis. Signaling can be both extra and intracellular, extracellular by means of for example receptors and intracellular by both canonical and non-canonical pathways (e.g., Wnt, AMPK, AKT signaling) [74, 75]. Studies of PLN that focus on identifying in what pathways the protein is involved and how the PLN mutant modulates signal transduction within the cell will allow us to identify specific agents that can be used to restore usual cell functions. This can be achieved by blocking or modulating aberrant signal transduction pathways in PLN R14del. As PLN works as an inhibitor of SERCA2a [15], overexpressing SERCA2a could be a promising strategy in which the disease may be alleviated. In the past, preclinical studies have shown that overexpressing SERCA2a was able to improve cardiac function [76, 77, 78, 79]. Unfortunately, in the CUPID 2 trial, where AAV1-mediated SERCA2a gene delivery in patients with HF was studied, no clinical benefit was found. The SERCA2a gene delivery, at the used dosage, was not able to reduce heart failure or terminal events when compared to placebo [80]. The lack of effect in this study was later attributed to lack of successful SERCA2a overexpression, due to a too low functional viral vector load caused by batch-to-batch variation in empty viral capsids [80, 81]. Due to this, SERCA2a overexpression may still be of benefit in PLN R14del or even general heart failure and should therefore be studied when it is known how to effectively deliver genes to the heart. Even though the results of the CUPID 2 trial yielded no success, it was an important step in the development of gene delivery related treatment strategies of cardiac disease.

In 2016, DWORF was identified [82], which is a promising new modulator of SERCA2a. DWORF is a transmembrane protein and a potent enhancer of SERCA2a activity, which does so by displacing PLN from SERCA2a [82, 83]. DWORF overexpression has shown to restore cardiac function and prevent pathological remodeling and Ca^2+^ dysregulation in a DCM mouse model [83]. If PLN R14del is proven to be a super inhibitor of SERCA2a, and DWORF is capable of preventing the interaction between SERCA2a and PLN, DWORF overexpression could be an attractive therapeutic for PLN R14del.

SERCA2a and DWORF overexpression are examples of PLN-related signal transduction modulation that show beneficial effects in heart disease that require fine tuning, but may be a promising therapeutic for future PLN R14del studies.

### Proposed Workflow of “Precision Medicine into practice”?

**Fig. 2:**
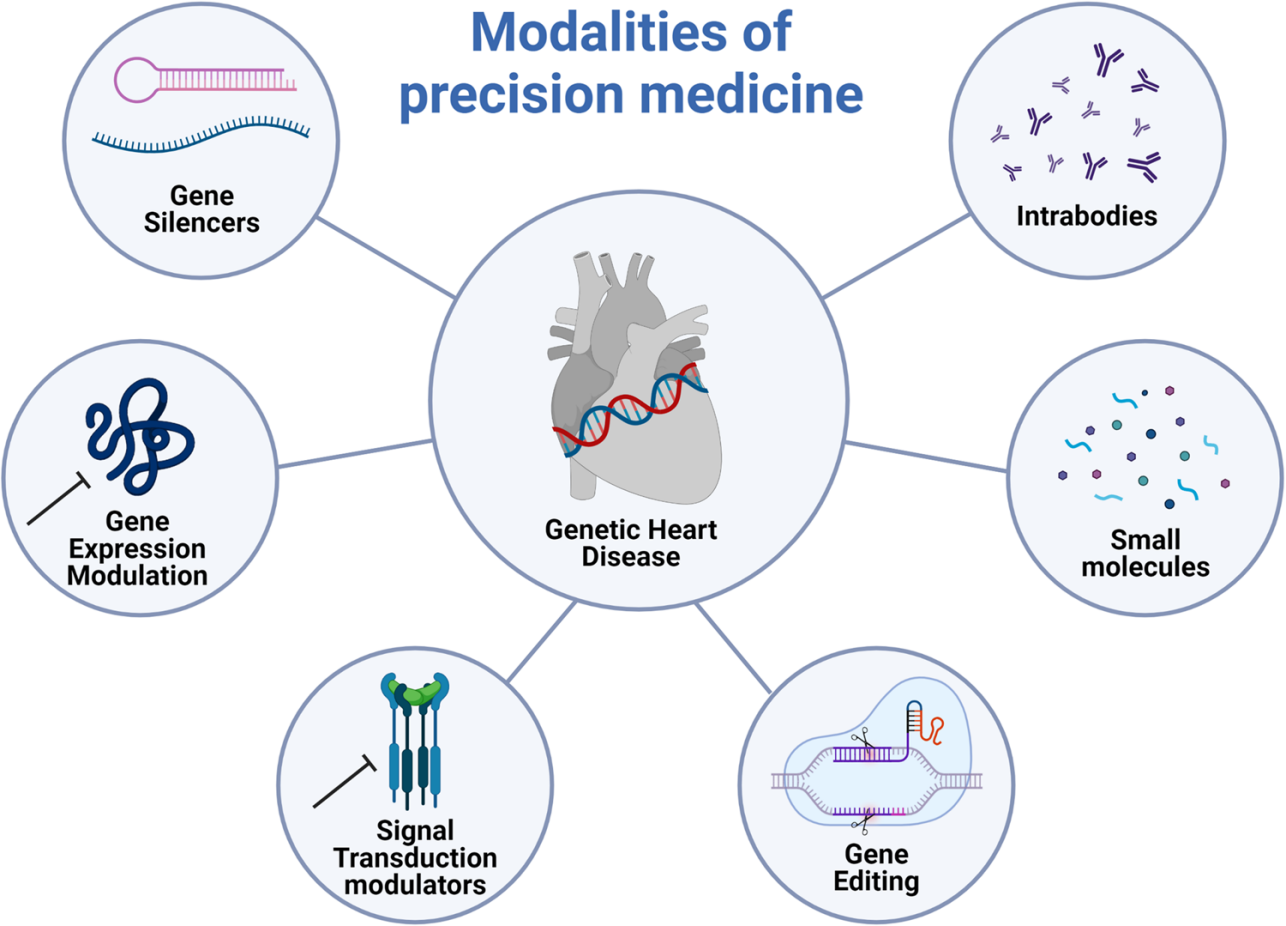
Modalities of precision medicine. Six modalities in precision medicine to be used for the treatment of genetic heart disease. Created with BioRender.com

**Fig. 3:**
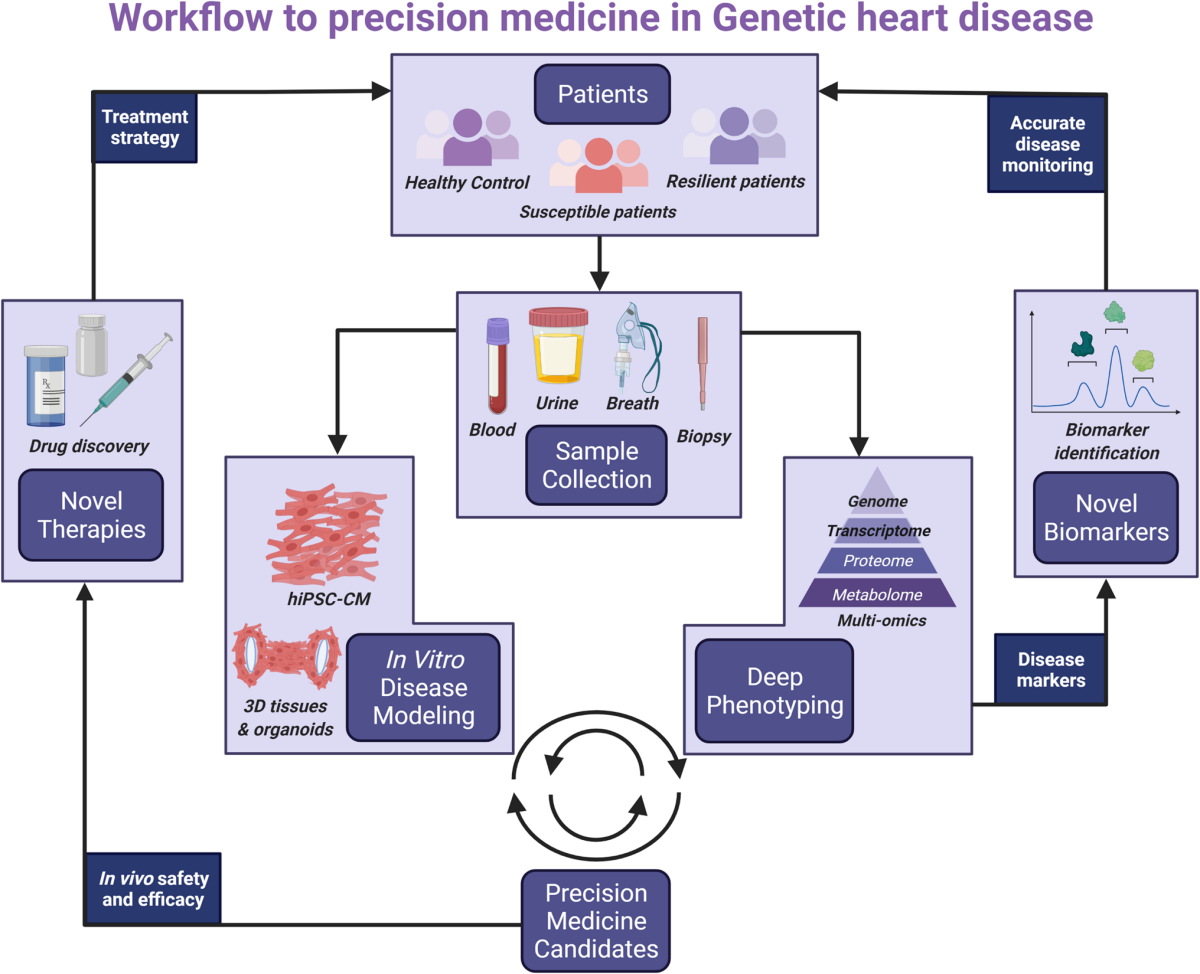
A workflow for precision medicine in genetic heart disease. A workflow based on deep phenotyping of patient-derived material and in vitro disease modeling. This will allow identification of therapeutic targets and disease modifiers, which will be used for the identification of novel biomarkers and the development of precision medicine. These biomarkers will allow accurate disease monitoring and precision medicine will allow the development of novel treatment strategies that taken together will ultimately help in optimizing patient care. Created with BioRender.com

PLN R14del is a great opportunity to optimize the workflow of precision medicine in genetic heart disease, due to the monogenetic nature and large patient population. We propose a workflow where patients are recruited and phenotyped in depth in order to identify disease modifiers and therapeutic targets that can be targeted using several modalities of precision medicine (Fig. 2). In this workflow, susceptible (age < 50 affected) and resilient (age > 50 unaffected) patients are recruited and matched with healthy controls (preferably family members). Patient materials (e.g., blood, urine, exhalation, tissue biopsies) are collected. Using reprogramming, patient-specific induced pluripotent stem cells (iPSCs) can be generated from skin tissue, blood, or urine [32]. Using gene-editing strategies such as CRISPR/Cas9, isogenic controls of patient lines can be generated and the phenotype can be corrected. These disease-specific and isogenic control iPSCs can be differentiated into hiPSC-CMs and studied to uncover disease pathophysiology and to test promising drugs that can ultimately make it to clinical trials (Fig. 3). Within this workflow, deep phenotyping includes multi-omics analysis and functional assays (electrophysiology, immunocytochemistry, metabolism, etc.). Detailed phenotyping of monogenetic disease remains necessary as long as we are not capable of efficiently and safely applying gene correction. In addition, phenotyping will allow us to understand the biological causes of heart disease and thereby identify patients at risk of disease development and progression, and monitor treatment effects.

Disease penetrance can be studied by recruiting patients that are susceptible or non-susceptible to disease development. Identifying which biological routes are differentially regulated between patients at different ends of the disease spectrum allows for the identification of risk factors contributing to the disease phenotype. Identifying biomarkers that correlate with disease is herein of utmost importance. Early identification of the disease onset in patients with a mendelian disease with an incomplete disease penetrance is of importance for the treatment of these patients. In the clinic, most yield is to be gained when a treatment is started at the moment of disease onset. Cardiac health is known to impact blood by altering inflammation, coagulation, redox balance, and the proteome [84]. Identifying which biomarkers in urine, blood, or exhalation correlate with disease state will allow for early diagnostics of disease and therefore more efficient treatment.

By identifying the causes of disease pathology, we can screen for drugs that are capable of restoring the mutation-induced alterations in the cardiomyocyte, which will help in disease alleviation and prevention.

### Future Perspectives

Precision medicine will play a major role in health care of the future, especially in the field of monogenetic heart disease where standard HF treatment options are insufficient and a population with common characteristics can easily be defined. The standardized one-size-fits-all approach to standard HF treatment helps to modulate symptoms of HF, but fails to address the core of the problem. Deep phenotyping of genetic heart disease followed by the development of precision medicine targeting disease modifiers will allow us to treat the core problem within a heart disease, such as the genetic mutation. Research will show what manners of precision medicine are safe to use and can be applied to efficiently treat patients burdened by their genetic background. In the future, the disease progression of patients with a genetic diagnosis can be monitored using disease-related biomarker screening. Upon onset of disease, effective precision medicine can be applied to alleviate disease and contribute to an increased health and lifespan.

## Conclusion

In this review, we highlight how precision medicine can be applied in the field of genetic cardiomyopathies. Genetic cardiomyopathies are a devastating disease, in which standard HF treatment options are not sufficient. In cases such as PLN R14del, patients can deteriorate despite their standardized treatment and will ultimately require a heart transplant or a left ventricular assist device. Understanding disease pathophysiology and identifying therapeutic targets will allow us to create precision medicine to help alleviate genetic heart disease. This can be achieved by, for example, modulating gene expression of the disease-causing genes or disease-associated signal transduction routes. The modalities discussed in this review are therapies that can be utilized as a treatment strategy for patients with genetic heart disease. With regard to PLN R14del-associated cardiomyopathy, the most promising modality of precision medicine is gene editing. Being capable of restoring a patient genetic background into a normal, harmless variant is the ultimate goal of precision medicine. Until then, other modalities that show promise such as PLN-ASOs and PLN siRNAs make great alternatives as a treatment strategy for genetic heart disease, as they have proven to be an effective treatment for PLN R14del and other etiologies of heart disease in vivo and in vitro [51, 52, 53, 54•]. In order to move these drugs towards a first clinical trial, in vivo safety and efficacy studies must first be performed in large mammals or non-human primates.

Using precision medicine, we can target specific pathophysiological processes that contribute to disease progression and thereby alleviate the disease phenotype. The most appropriate drug for an individual patient genotype may then be selected from an existing panel. Due to the relatively high prevalence of PLN R14del-associated cardiomyopathy in the Netherlands, many studies are currently performed to identify disease modifiers and pathophysiology, and what can be done to prevent disease development. These studies performed in favor of the PLN R14del-associated cardiomyopathy patient population will help translate precision medicine to more rare genetic disease as well. Identifying which treatment strategies are capable of efficiently targeting and restoring the heart will also make promising treatment strategies for other genetic heart disease, beyond PLN R14del.
